# No effect of beetroot juice supplementation on exercise economy and performance in recreationally active females despite increased torque production

**DOI:** 10.14814/phy2.13982

**Published:** 2019-01-17

**Authors:** Kate A. Wickham, Devin G. McCarthy, Jamie M. Pereira, Daniel T. Cervone, Lex B. Verdijk, Luc J. C. van Loon, Geoffrey A. Power, Lawrence L. Spriet

**Affiliations:** ^1^ Department of Human Health and Nutritional Sciences University of Guelph Ontario Canada; ^2^ Department of Human Biology Maastricht University Maastricht The Netherlands

**Keywords:** Beetroot juice, contractile properties, females, torque‐frequency, oxygen uptake, performance

## Abstract

This study investigated the effects of acute and chronic beetroot juice (BRJ) supplementation on submaximal exercise oxygen uptake (VO_2_), time trial (TT) performance, and contractile properties of the plantar flexors in females. Study 1: Using a double blind, randomized, crossover design, 12 recreationally active females using hormonal contraceptives supplemented acutely (2.5 h) and chronically (8 days) with 280 mL BRJ/d (~26 mmoles nitrate [${\text{NO}}_{3}^{-}$]) or a ${\text{NO}}_{3}^{-}$‐free placebo (PLA). On days 1 and 8, participants cycled for 10 min at 50% and 70% VO_2peak_ and completed a 4 kJ/kg body mass TT. Plasma [${\text{NO}}_{3}^{-}$] and nitrite ([NO_2_^−^]) increased significantly following BRJ supplementation versus PLA. There was no effect of BRJ supplementation on VO_2_ at 50% or 70% VO_2peak_, or TT performance. Study 2: 12 recreationally active females (*n* = 7 from Study 1) using hormonal contraceptives participated in a baseline visit and were supplemented acutely (2.5 h) and chronically (8 days) with 280 mL BRJ/d. Maximum voluntary strength (MVC) of the plantar flexors was assessed and a torque‐frequency curve performed. BRJ had no effect on MVC, voluntary activation, peak twitch torque, time to peak torque, or half relaxation time. Following both acute (46.6 ± 4.9% of 100 Hz torque) and chronic (47.2 ± 4.4%) supplementation, 10 Hz torque was significantly greater compared to baseline (32.9 ± 2.6%). In summary, BRJ may not be an effective ergogenic aid in recreationally active females as it did not reduce submaximal exercise VO_2_ or improve aerobic TT performance despite increasing low frequency torque production.

## Introduction

Nitric oxide (NO) is a potent signaling molecule that may play a pivotal role in the regulation of several biological processes including vasodilation (Ignarro et al. 1987), mitochondrial efficiency (Larsen et al. 2011) and excitation‐coupling (Bailey et al. 2010; Haider and Folland 2014; Whitfield et al. 2016, 2017). NO can be generated via two distinct pathways, endogenously through the classic oxygen (O_2_) dependent l‐arginine/NO synthase pathway and exogenously through the O_2_ independent nitrate (${\text{NO}}_{3}^{-}$)‐nitrite (NO_2_^−^)‐NO pathway (Bailey et al. 2012). The latter pathway is facilitated by commensal anaerobic bacteria in the oral cavity that sequentially reduce ${\text{NO}}_{3}^{-}$ to NO_2_^−^ and further to NO (Bailey et al. 2012).

The last decade has seen a plethora of research investigating the physiologic and metabolic effects of dietary ${\text{NO}}_{3}^{-}$ supplementation. Beetroot juice (BRJ) has been at the forefront of this research due to its high dietary ${\text{NO}}_{3}^{-}$ content (Hord et al. 2009) and has demonstrated effectiveness for general health as well as athletic performance (Bailey et al. 2012). On the athletic front, BRJ supplementation has been shown to reduce the O_2_ cost of submaximal exercise and ultimately improve exercise performance in recreationally trained males both acutely and chronically (Bailey et al. 2009; Lansley et al. 2010; Vanhatalo et al. 2010).

Although the exact mechanism(s) of action has yet to be resolved, recent evidence suggests that dietary ${\text{NO}}_{3}^{-}$ supplementation may reduce the O_2_ cost of exercise by decreasing the ATP cost of skeletal muscle contraction (Bailey et al. 2010; Hernandez et al. 2012; Haider and Folland 2014; Whitfield et al. 2017; Coggan and Peterson 2018). The major ATP costs accrued during skeletal muscle contraction are by myosin ATPase and sarcoendoplasmic reticulum calcium ATPase (SERCA) (Barclay et al. 2007). Recently, Whitfield et al. (2017) demonstrated an increase in skeletal muscle force production with low frequency stimulation at 10 Hz with no difference in the expression of calcium‐handling proteins following chronic BRJ supplementation. These results may indicate the role of post‐translational modifications of contractile proteins following BRJ supplementation, and begs the question, whether acute BRJ supplementation can also enhance low frequency torque production.

Despite a growing body of evidence supporting the ergogenic effects of BRJ supplementation in recreationally active males, females are heavily underrepresented in this field of research. Vanhatalo et al. (2010) was the first group to demonstrate a reduction in the O_2_ cost of exercise in a cohort including recreationally active female participants (five male, three female). To date, only two published studies have attempted to determine the effects of BRJ supplementation on the O_2_ cost of submaximal exercise in strictly non‐elite female participants (Bond et al. 2014; Rienks et al. 2015). Bond et al. (2014) found a significant reduction in the O_2_ cost of submaximal cycling at three different exercise intensities in sedentary females following acute BRJ supplementation. However, these findings are difficult to interpret due to the ambiguous reporting of the VO_2_ data (i.e. only graphical presentation of relative VO_2_) and the lack of a ${\text{NO}}_{3}^{-}$‐free placebo treatment. It is also difficult to extrapolate these findings in a sedentary population to recreationally active individuals. Rienks et al. (2015) demonstrated a reduction in the O_2_ cost of cycling with acute BRJ supplementation during a 75 W cooldown following no change in performance during a 20 min rating of perceived exertion (RPE) clamp protocol in recreationally active females. It is difficult to make clear interpretations of these results as the VO_2_ protocol does not accurately validate the effects of BRJ supplementation on submaximal exercise economy and there were no measurements of plasma ${\text{NO}}_{3}^{-}$ and ${\text{NO}}_{2}^{-}$ levels prior to or following supplementation. Moreover, no study has (1) investigated the effects of chronic BRJ supplementation in non‐elite females, (2) investigated both acute and chronic BRJ supplementation in females within the same study protocol, and (3) assessed skeletal muscle contractile properties in an exclusively female population following BRJ supplementation.

Interestingly, the beneficial effects of dietary ${\text{NO}}_{3}^{-}$ supplementation on submaximal VO_2_ do not appear to be present in elite males (Boorsma et al. 2014; Nyakayiru et al. 2017a) and females (Lane et al. 2014). It is proposed that these individuals have greater NO availability and enhanced exercise economy compared to recreationally active individuals which may negate the positive effects of dietary ${\text{NO}}_{3}^{-}$ supplementation (Porcelli et al. 2015).

Therefore, the aim of the current study was to determine if acute and chronic BRJ supplementation reduced the O_2_ cost of submaximal exercise, improved aerobic TT performance, and improved skeletal muscle torque production and indices of contractile function in recreationally active females. We hypothesized that both acute and chronic BRJ supplementation would reduce the O_2_ cost of submaximal exercise and improve aerobic TT performance, as this has been consistently demonstrated in recreationally active males. Furthermore, we hypothesized that acute and chronic BRJ supplementation would improve skeletal muscle torque production with low frequency stimulation, as this has also been demonstrated with chronic supplementation in recreationally active males.

## Methods

### Subjects

Twelve recreationally active females were recruited to participate in the O_2_ cost and performance study (mean ± SD, age: 23 ± 1 year; weight: 65.0 ± 10.0 kg; height: 1.69 ± 0.06 m; VO_2peak_: 40.7 ± 4.3 mL/kg/min). Subjects were included in the study if they were healthy, recreationally active females, between the ages of 18 and 30, using hormonal contraceptives for at least 6 months prior to study participation, and consumed less than 300 mg dietary ${\text{NO}}_{3}^{-}$ per day. The recreationally active subjects (VO_2peak_, 35–45 mL/kg/min) reported being physically active 4 ± 1 day/week and performed 4.5 ± 1.5 h exercise/week in the 6 months prior to the study. Seven of the participants from Study 1 returned for the contractile properties study and an additional five subjects were recruited (*n* = 12, age: 22 ± 2 year; weight: 65.6 ± 8.8 kg; height: 1.69 ± 0.06 m; VO_2peak_: 41.5 ± 4.1 mL/kg/min). The participants were informed of the study requirements and potential risks prior to providing oral and written consent. All procedures were approved by the University of Guelph Research Ethics Board and conformed to the Declaration of Helsinki.

### Study overview

The O_2_ cost and performance study comprised seven laboratory visits each separated by ~1 week. The first visit consisted of a VO_2peak_ test, with familiarization trials performed during visits two and three. For the final four visits, subjects ingested 280 mL/d of either BRJ (2 × 6.5–mmol ${\text{NO}}_{3}^{-}$/70 mL Beet It Sport, taken twice daily for a total intake of ~26 mmol ${\text{NO}}_{3}^{-}$; James White Drinks, Ipswich, United Kingdom) or a ${\text{NO}}_{3}^{-}$‐free placebo (2 × 70 mL, taken twice daily; James White Drinks, Ipswich, United Kingdom) for 8 days and were tested on days 1 and 8 using a double‐blind, randomized, counter‐balanced, crossover design. A 9 ± 1 day washout period separated the supplementation periods. The contractile study was performed immediately following the O_2_ cost and performance study and comprised four laboratory visits each separated by ~1 week. The first visit consisted of a familiarization to the electrical stimulation protocol. During the second visit baseline measurements were made and visits three and four were the acute (1 day) and chronic (8 days) BRJ supplementation trials, which followed the same BRJ supplementation protocol as the first study.

### O_2_ cost and performance study

#### VO_2peak_ test and familiarization trials

To determine VO_2peak_, and the cycling intensity (i.e. watts) to elicit 50 and 70% VO_2peak_, each subject performed an incremental exercise test to volitional exhaustion on a cycle ergometer (Lode Excalibur Sport, Lode, Groningen, The Netherlands). The subjects were equipped with a heart rate (HR) monitor (Polar H10, Polar, Oulu, Finland) and HR, VO_2_, and power output (PO) were recorded at the end of each step increment. VO_2_ and carbon dioxide production (VCO_2_) were measured (Moxus Modular VO_2_ System, AEI Technologies, Pittsburgh, USA) in 20 sec intervals.

The subjects completed two familiarization trials prior to the experimental trials. In the first familiarization trial the subjects were set up comfortably on the ergometer and the position was documented for subsequent trials. During the familiarization trials, the subjects completed the entire experimental exercise protocol consisting of a 5 min warm up at 60 W, 10 min at 50% VO_2peak_, 10 min at 70% VO_2peak_, and a 4 kJ/kg body mass (BM) aerobic timebased TT. In the first familiarization trial the subjects consumed water ad libitum. The subjects were weighed before and after the trial for determination of BM loss through sweat. If the subject lost or gained BM compared to the initial weigh in, then the volume of fluid provided during the exercise protocol was adjusted prior to the second familiarization trial. The volume of water consumed in the second familiarization trial was replicated for the subsequent experimental trials. The subjects moved to the experimental trials once there was less than 5% variability between the TT performances (Currell and Jeukendrup 2008). The variability of the subjects’ familiarization TT performances was less than 5% (3.4 ± 2.1%: Familiarization 1, 1760 ± 102; Familiarization 2, 1798 ± 96 sec), therefore no subject was required to complete a third familiarization trial.

The subjects recorded their dietary intake, sleep, and exercise in the 48 h before the familiarization and experimental trials and were asked to replicate their diet, sleep, and exercise habits for the duration of the study. In the 24 h prior to the familiarization and experimental trials, the subjects were asked to refrain from intense exercise and alcohol, and to abstain from caffeine for 6 h before the trials.

#### Experimental trials

The use of antibacterial mouthwash and chewing gum was prohibited during the experimental trials, as there is evidence suggesting that these substances destroy the anaerobic bacteria in the mouth necessary for the conversion of ${\text{NO}}_{3}^{-}$ to NO_2_^−^ and NO (McDonagh et al. 2015). Furthermore, the subjects were instructed to avoid brushing their teeth for 2 h before and after the ingestion of the BRJ or placebo (PLA).

For the experimental trials, the subjects arrived at the laboratory after an overnight fast. A baseline blood sample (9.5 mL) was drawn for analysis of serum estrogen and progesterone as well as plasma [${\text{NO}}_{3}^{-}$] and [${\text{NO}}_{2}^{-}$]. The subjects then consumed a standardized breakfast within 15 min, consisting of a banana, a SoLo GI® bar (SoLo GI Nutrition, Kelowna, Canada), and 140 mL of BRJ or PLA. This breakfast was intentionally carbohydrate (CHO) rich to ensure that CHO was not limiting during exercise (79 g CHO, 16.5 g protein, 7.7 g fat). Following the standardized breakfast, the subjects were required to wait for 2 h before a second blood sample was drawn (6 mL). During this period, the subjects were provided with water to ensure adequate hydration prior to exercise. The volume of water consumed at rest in the first experimental trial was replicated for subsequent trials.

Immediately after the second blood sample, the subjects were escorted to the exercise laboratory (mean ± SD: Temp; 21.4 ± 1.0°C, Humidity; 56.0 ± 10.4%). The subjects were asked to provide a urine sample for determination of hydration status via urine specific gravity (USG) and to void their bladder. USG was determined using a pen refractometer (Atago 3730, Atago CO., LTD., Tokyo, Japan). Then, the subjects were weighed and equipped with a HR monitor. Exercise testing commenced ~2.5 h post‐beverage ingestion ensuring that peak plasma ${\text{NO}}_{2}^{-}$ levels were achieved (Wylie et al. 2013). The subjects completed a 5 min warmup at 60 W followed by 10 min at 50% VO_2peak_ (81 ± 13 W) and 10 min at 70% VO_2peak_ (131 ± 17 W) and were instructed to maintain a cadence of 80 revolutions per min (rpm) throughout the submaximal cycling protocol. The subjects then rested for 3 min before completing a 4 kJ/kg BM TT (260 ± 43 kJ) as fast as possible. The ergometer was set to linear mode, and the subjects started cycling at a resistance equal to 70% VO_2peak_ at 80 rpm. The subjects were able to increase the PO by increasing their rpms and were only shown the number of kJ completed during the TT.

RPE was recorded every 5 min throughout the submaximal exercise protocol using the Borg 20 Scale (Borg 1982). Respiratory measurements were recorded over 20 sec intervals for the final 4 min of each submaximal exercise intensity. Time elapsed, PO, rpm and RPE were recorded and verbal encouragement was given with the completion of each 20% of the TT. HR was recorded continuously throughout the exercise protocol. Following the TT, subjects completed a blinding and side effects questionnaire.

#### Blood analyses

Morning blood samples were collected in serum separator tubes (3.5 mL) for determination of serum estrogen and progesterone, and K_2_EDTA‐coated tubes (6 mL) for determination of plasma ${\text{NO}}_{3}^{-}$ and ${\text{NO}}_{2}^{-}$. A second blood sample (6 mL) was collected 2 h post‐ingestion. Immediately following collection, the K_2_EDTA‐coated tubes were centrifuged for 10 min at 10,000 *g* and 4°C. The serum separator tubes were left for 30 min at room temperature to coagulate and were centrifuged for 10 min at 12,000 *g* and 4°C. Plasma and serum aliquots were frozen and stored at ‐80°C for future analyses.

Serum samples were analyzed for estradiol (pg/mL) and progesterone (ng/mL) concentrations using commercially available ELISA kits (Abcam, Item Nos: ab108667, ab108654, Cambridge, United Kingdom). Plasma samples were analyzed for ${\text{NO}}_{3}^{-}$ and ${\text{NO}}_{2}^{-}$ concentrations by chemiluminescence using a Sievers gas‐phase chemiluminescence NO analyzer (Sievers NOA 280i; Analytix, Durham, United Kingdom) (Lundberg and Govoni 2004).

### Contractile properties study

#### Familiarization and experimental trials

The participants were asked to adhere to the same pre‐testing protocol as the O_2_ cost and performance study, and the same acute and chronic BRJ supplementation protocol was utilized. No PLA treatment was used during this experiment, as the primary outcome was electrically‐evoked skeletal muscle contractions, which are unaffected by the volition of the subjects and the blinding process.

A HUMAC NORM dynamometer (CSMi Medical Solutions, Massachusetts, USA) was used for data collection. A footplate was attached to the dynamometer and the right foot was strapped tightly to the footplate with the lateral malleolus in line with the rotational axis of the dynamometer. Great care was taken to minimize heel lift during contraction. If the heel slipped or visible lift occurred, the trial was repeated. Participants were instructed to “tip the plate over” using the calf muscles and not extend or flex the knee. Subjects were secured to minimize extraneous body movements with an adjustable 4‐point inelastic harness and the right leg was secured further with a Velcro strap running over the thigh. Participants sat in a slightly reclined position with knee and ankle angles at 90° and 10° plantar flexion respectively to minimize torque contribution from the gastrocnemius muscle (Dalton et al. 2013, 2018). A computer‐triggered stimulator (Model DS7A, Digitimer, Welwyn Garden City, United Kingdom) provided the electrical stimulation of the plantar flexors via a bar electrode positioned over the tibial nerve. The pulse width was 1000 *μ*sec.

Peak twitch torque was determined by increasing the amplitude of the current until a plateau in torque was reached followed by a further 10–15% increase in current to ensure supramaximal stimulation. This current was then used to assess voluntary activation (VA) (i.e. interpolated twitch technique) during maximum voluntary contractions (MVC). Participants performed three MVC (3–5 sec) each separated by 3 min rest, with a twitch delivered manually prior to, during the peak plateau (SIT), and immediately succeeding (RT) the MVC. The interpolated twitch technique (Hales and Gandevia 1988) was used to ensure near complete voluntary activation during all maximal efforts and was calculated as VA = [1 − (SIT/RT)] × 100%. During all maximal efforts, participants were encouraged verbally and instructed to contract as hard and fast as possible and then hold until instructed to relax their plantar flexors fully. A computer monitor displayed the torque output during all contractions. A true MVC was accepted when there was less than 5% variation in MVC torque, and VA was >90%. The “pre” twitch data was used for determination of peak twitch torque, time to peak torque, and half relaxation time. Next, the current needed to elicit 30% MVC at 100 Hz was identified by increasing the current until the target torque was achieved. A torque‐frequency curve was constructed by using 1 sec trains of the following frequencies: 1, 5, 10, 20, 30, 40, 50, and 100 Hz. One min rest was given between each stimulation. Torque values for the MVC and evoked contractions are reported as the peak. Although the plantar flexors are not the main muscle group used during cycling, this lower limb muscle group was examined due to the ease and accuracy of the torque‐frequency curve generated from this muscle.

### Compliance

Each subject's adherence to the chronic supplementation protocol was assessed through plasma ${\text{NO}}_{3}^{-}$ and ${\text{NO}}_{2}^{-}$ analysis.

### Statistics

Diet records were analyzed using Food Processor Nutrition Analysis Software (ESHA Research, Oregon, USA). Statistical analyses were performed using Prism 7 (GraphPad Software, San Diego, USA). Statistical significance was achieved when *P* < 0.05. Data are presented as mean ± SD unless otherwise stated. The statistical tests for serum estradiol and progesterone, pre‐trial characteristics (48 h diet and exercise record, pre‐trial sleep), environmental conditions (temperature, humidity), hydration measures (pre‐fluid intake, USG, during‐fluid intake), and submaximal exercise economy (VO_2_, HR and RPE) were conducted using a two‐way analysis of variance (ANOVA) and Tukey's multiple comparisons post hoc test. Time trial performance data (HR, RPE, PO, rpm, and elapsed time) were analyzed using a three‐way ANOVA (% complete × condition × supplement) and Tukey's multiple comparisons post hoc test.

All contractile data were analyzed using LabChart software (LabChart, Pro Modules 2015, Version 8). MVC, twitch peak torque, time to peak torque, half relaxation, and torque at 100 Hz were analyzed using a one‐way ANOVA. The torque‐frequency curve was analyzed using a two‐way ANOVA and Tukey's multiple comparisons post hoc test.

## Results

### O_2_ cost and performance study

The subjects were 100% compliant to the supplementation protocol as assessed through plasma ${\text{NO}}_{3}^{-}$ and ${\text{NO}}_{2}^{-}$ analysis. The results from the blinding and side effects questionnaire indicated that 100% of the subjects were successfully blinded to the treatments they received, and 58% of the subjects experienced side effects with acute and chronic supplementation (BRJ and PLA). Mild nausea was the most commonly reported side effect (41%), while 17% reported gastrointestinal upset, 17% reported beeturia, and 8% reported acid reflux.

### Control parameters

There was no difference in the 48 h dietary intake between conditions (Acute PLA, 2447 ± 1027; Chronic PLA, 2150 ± 510; Acute BRJ, 2155 ± 932; Chronic BRJ, 2316 ± 879 kcal/day) and no difference in the mean number of hours slept between trials (Acute PLA, 7.6 ± 0.8; Chronic PLA, 7.3 ± 0.8; Acute BRJ, 7.4 ± 0.6; Chronic BRJ, 7.2 ± 1.0 h). The subjects were well hydrated prior to exercise and USG was not different between conditions (Acute PLA, 1.003 ± 0.002; Chronic PLA, 1.005 ± 0.006; Acute BRJ, 1.005 ± 0.004; Chronic BRJ, 1.005 ± 0.004). The subjects consumed the same volume of fluid prior to each trial (Acute PLA, 677 ± 150; Chronic PLA, 674 ± 163; Acute BRJ, 658 ± 178; Chronic BRJ, 651 ± 200 mL) as well as during each exercise trial (Acute PLA, 576 ± 195; Chronic PLA, 558 ± 198; Acute BRJ, 584 ± 216; Chronic BRJ, 560 ± 188 mL). There was no difference in the change in BM post‐exercise between conditions (Acute PLA, −0.1 ± 0.5; Chronic PLA, −0.2 ± 0.3; Acute BRJ, −0.2 ± 0.5; Chronic BRJ, −0.1 ± 0.3%) indicating that the subjects were adequately hydrated throughout the exercise protocol.

### Serum estradiol and progesterone

Serum estradiol concentrations were not significantly different across any of the trials (Acute PLA, 0.01 ± 0.01; Chronic PLA, 0.01 ± 0.02; Acute BRJ, 0.02 ± 0.02; Chronic BRJ, 0.01 ± 0.02 pg/mL) (*n* = 10). Serum progesterone concentrations were not significantly different across any of the trials (Acute PLA, 0.98 ± 1.13; Chronic PLA, 1.39 ± 3.08; Acute BRJ, 0.47 ± 0.42; Chronic BRJ, 1.14 ± 2.39 ng/mL) (*n* = 10).

### Plasma [${\text{NO}}_{3}^{-}$] and [${\text{NO}}_{2}^{-}$]

**Figure 1:**
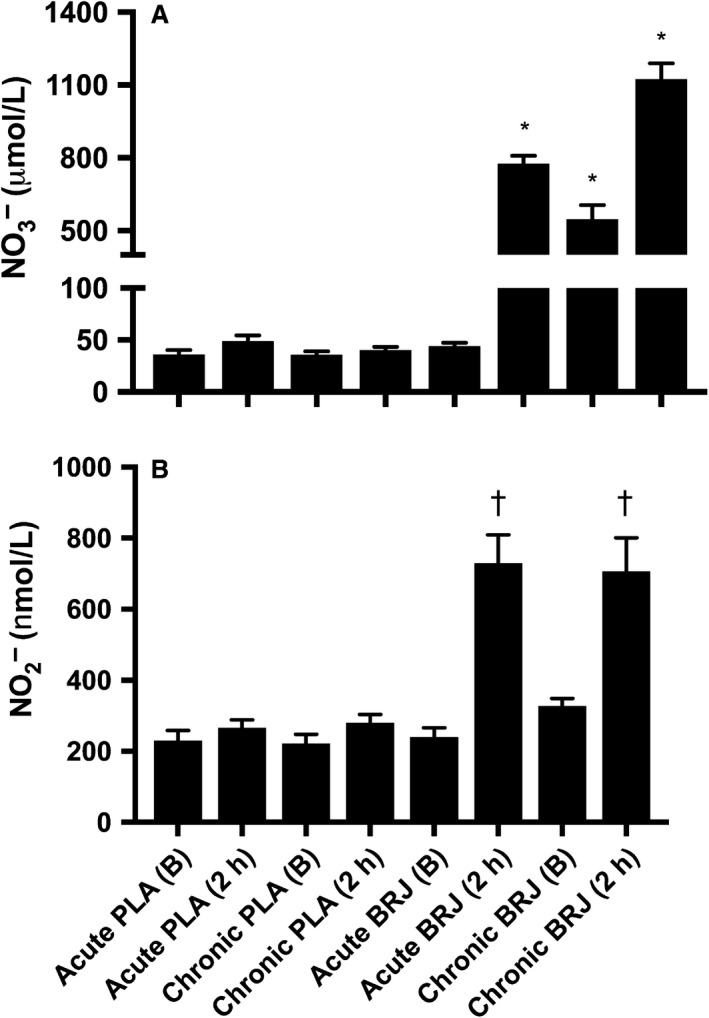
(A) Plasma nitrate (${\text{NO}}_{3}^{-}$) at baseline (B) and two hours post‐ingestion (2 h) of 140 mL beetroot juice (BRJ) or nitrate‐free placebo (PLA) both acutely and chronically. (B) Plasma nitrite (${\text{NO}}_{2}^{-}$) at B and 2 h of 140 mL BRJ or PLA both acutely and chronically.Values are mean ± SE,* n* = 12. *Significantly different than all other conditions. ^†^Significantly greater than conditions with no symbol.

Baseline plasma ${\text{NO}}_{3}^{-}$ concentrations were not different in the Acute PLA (36 ± 14 *μ*mol/L), Chronic PLA (36 ± 11 *μ*mol/L), and Acute BRJ trials (44 ± 10 *μ*mol/L) (Fig. 1A). However, plasma ${\text{NO}}_{3}^{-}$ was significantly elevated at baseline in the chronic BRJ trial (548 ± 189 *μ*mol/L) compared to Acute PLA, Chronic PLA, and Acute BRJ. There was no significant difference in plasma ${\text{NO}}_{3}^{-}$ 2 h post‐beverage ingestion in the Acute PLA (49 ± 18 *μ*mol/L) and Chronic PLA (40 ± 10 *μ*mol/L) trials. Conversely, there was a significant rise in plasma ${\text{NO}}_{3}^{-}$ concentrations 2 h post‐beverage ingestion in the Acute BRJ (776 ± 110 *μ*mol/L) and Chronic BRJ (1125 ± 215 *μ*mol/L) trials. Plasma ${\text{NO}}_{3}^{-}$ concentrations were significantly greater in the Acute BRJ trial compared to Acute PLA and Chronic PLA 2 h post‐beverage consumption. Similarly, 2 h post‐beverage consumption, plasma ${\text{NO}}_{3}^{-}$ concentrations were significantly greater in the Chronic BRJ trial compared to Acute PLA and Chronic PLA. Interestingly, plasma ${\text{NO}}_{3}^{-}$ concentrations were significantly greater in the Chronic BRJ compared to the Acute BRJ trial 2 h post‐beverage consumption.

Plasma ${\text{NO}}_{2}^{-}$ concentrations were not significantly different at baseline across all trials (Acute PLA, 231 ± 96; Chronic PLA, 216 ± 89; Acute BRJ, 240 ± 90; Chronic BRJ, 328 ± 70 nmol/L) (Fig. 1B). There was no change in plasma ${\text{NO}}_{2}^{-}$ concentrations 2 h post‐beverage ingestion in the Acute PLA (266 ± 79 nmol/L) and Chronic PLA (280 ± 83 nmol/L) trials. The rise in plasma ${\text{NO}}_{2}^{-}$ 2 h post‐beverage ingestion was significant in both the Acute BRJ (729 ± 277 nmol/L) and Chronic BRJ (706 ± 329 nmol/L) trials, but there was no difference between these conditions.

### Submaximal exercise economy

**Figure 2:**
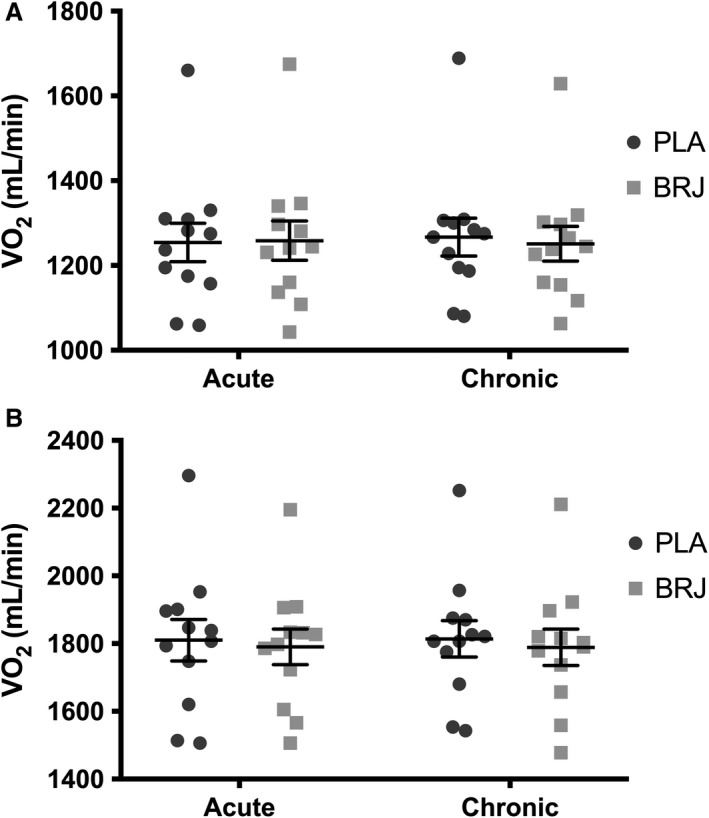
(A) Mean oxygen uptake (VO_2_) at 50% peak oxygen uptake (VO_2peak_) following acute and chronic supplementation with beetroot juice (BRJ) or a nitrate‐free placebo (PLA). (B) Mean VO_2_ at 70% VO2peak following acute and chronic supplementation with BRJ or PLA. Data reported as mean ± SE,* n* = 12.

There was no change in mean VO_2_ between the trials when the subjects cycled at an intensity that elicited 50% VO_2peak_ (Acute PLA, 1254 ± 157; Chronic PLA, 1267 ± 155; Acute BRJ, 1259 ± 161; Chronic BRJ, 1252 ± 144 mL/min O_2_) (Fig. 2A). There was no difference in HR at 5 or 10 min between conditions at 50% VO_2peak_ (Table 1). RPE was not significantly different between conditions, however there was a main effect of time where RPE was significantly increased from 5 to 10 min at 50% VO_2peak_ (*P* < 0.0001) (Table 1). Similarly, mean VO_2_ was unchanged at 70% VO_2peak_ between trials (Acute PLA, 1804 ± 209; Chronic PLA, 1814 ± 186; Acute BRJ, 1791 ± 182; Chronic BRJ, 1789 ± 186 mL/min O_2_) (Fig. 2B). There was no difference in HR at 5 or 10 min between conditions at 70% VO_2peak_ (Table 1). RPE was not significantly different between conditions, however there was a main effect of time where RPE increased from 5 to 10 min at 70% VO_2peak_ (*P* < 0.0001) (Table 1).

**Table 1:** Heart rate (HR) and rating of perceived exertion (RPE) (rpm) recorded at 5 and 10 min at 50% and 70% peak oxygen consumption (VO_2peak_) following acute and chronic supplementation with beetroot juice (BRJ) or a nitrate‐free placebo (PLA)

		50% VO_2peak_		70% VO_2peak_	
		5 min	10 min	5 min	10 min
HR (bpm)	Acute PLA	132 ± 14	133 ± 15	160 ± 15	164 ± 15
	Chronic PLA	133 ± 10	131 ± 10	160 ± 10	165 ± 11
	Acute BRJ	133 ± 11	133 ± 13	160 ± 13	164 ± 14
	Chronic BRJ	134 ± 11	132 ± 12	160 ± 13	163 ± 14
RPE	Acute PLA	9.6 ± 2.0	10.1 ± 1.8*	12.6 ± 0.8	13.5 ± 0.8*
	Chronic PLA	9.8 ± 1.9	10.7 ± 0.5*	12.6 ± 0.9	13.8 ± 0.9*
	Acute BRJ	10.2 ± 1.7	10.2 ± 1.7*	12.4 ± 1.4	13.5 ± 1.2*
	Chronic BRJ	9.3 ± 2.2	10.3 ± 2.3*	12.3 ± 1.5	13.5 ± 1.3*

Values reported as mean ± SD, *n* = 12.

* Significant increase from 5 to 10 min (main effect of time).

### Time trial performance

**Figure 3:**
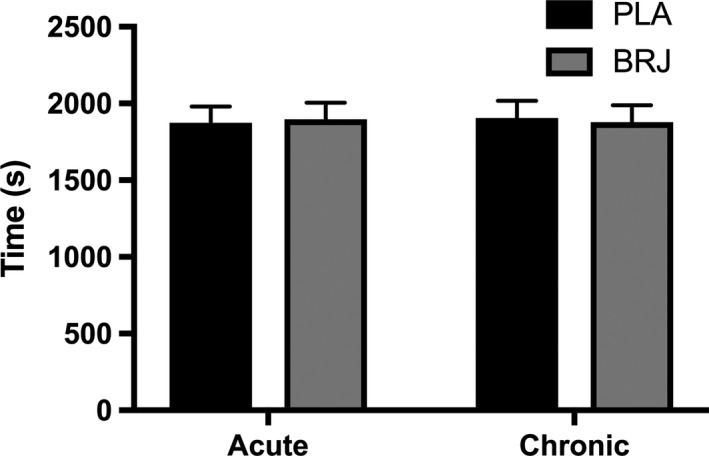
Mean time trial completion following acute and chronic supplementation with beetroot juice (BRJ) or a nitrate‐free placebo (PLA). Values reported as mean ± SE,* n* = 12.

**Figure 4:**
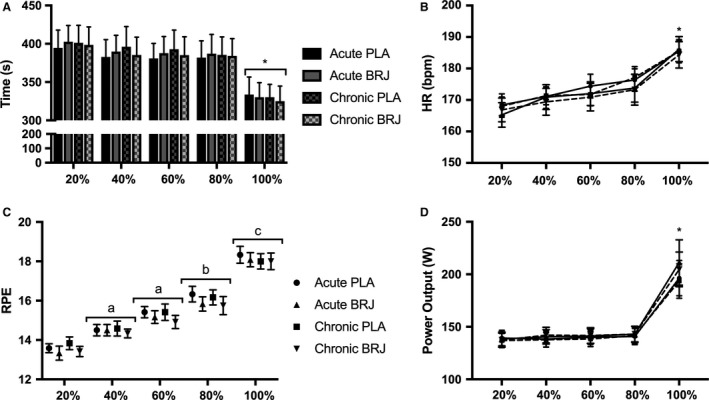
Time trial performance parameters following acute and chronic supplementation with beetroot juice (BRJ) or a nitrate‐free placebo (PLA). (A) Mean time to complete each 20% split (B) Mean heart rate (HR) at each 20% split. (C) Mean rating of perceived exertion (RPE) at each 20% split. (D) Mean power output at each 20% split. Values reported as mean ± SE,* n* = 12. *Significantly greater than all other time points; a, significantly greater than 20%; b, significantly greater than 20%, 40% and 60%; c, significantly greater than 20, 40, 60 and 80%.

There was no significant difference in TT completion across the trials (Acute PLA, 1845 ± 247; Chronic PLA, 1869 ± 259; Acute BRJ, 1912 ± 322; Chronic BRJ, 1892 ± 339 sec) (Fig. 3). There was also no learning effect associated with TT completion (Trial 1, 1857 ± 356; Trial 2, 1873 ± 372; Trial 3, 1913 ± 387; Trial 4, 1912 ± 399 sec). Furthermore, there was no difference in the time to complete each 20% split between trials. There was a main effect of time in which the final split was significantly faster than the other splits (*P* = 0.0001) (Fig. 4A). No difference was observed for HR throughout the TT. However, a main effect of time was demonstrated, where HR was significantly higher in the final split compared to the rest of the TT (*P* < 0.0001) (Fig. 4B). There was no significant effect of condition on RPE, but there was a main effect of time resulting in a progressive, significant rise in RPE throughout the TT (*P* < 0.0001) (Fig. 4C). Additionally, there was a trend for decreased RPE with BRJ compared to PLA (*P* = 0.07). There was no significant difference in rpm or PO between conditions during the TT. However, there was a main effect of time in which rpm (*P* < 0.0001) and PO (*P* < 0.0001) were significantly increased in the final split compared to the rest of the TT (Fig. 4D).

#### Contractile properties study

The results from the side effects questionnaire indicated that 33% of the subjects experienced side effects with acute and chronic supplementation. Mild nausea was the most commonly reported side effect (25%) and 8% reported gastrointestinal upset.

### Contractile properties

**Figure 5:**
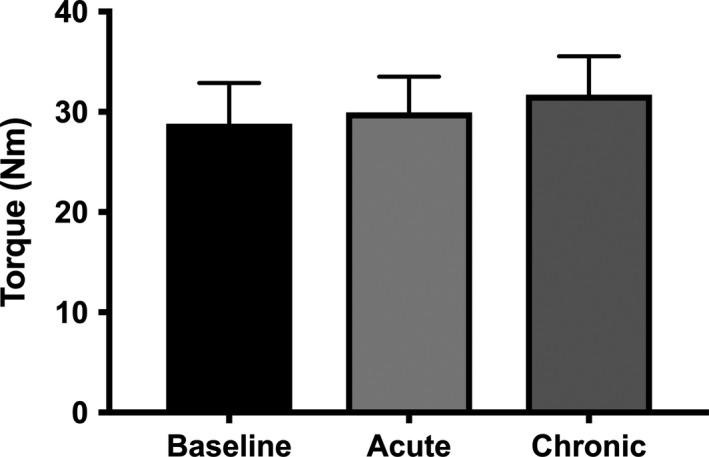
Absolute torque produced with 100 Hz stimulation during the torque‐frequency curve of the plantar flexors following stimulation of the tibial nerve at baseline and following acute and chronic BRJ supplementation. Values reported as mean ± SE,* n* = 12.

**Figure 6:**
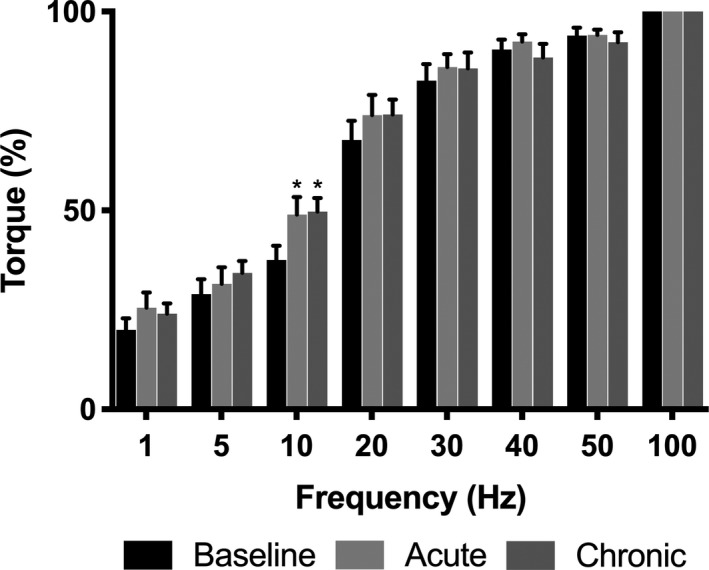
Torque‐frequency curve of the plantar flexors following stimulation of the tibial nerve at baseline and following acute and chronic BRJ supplementation. Data normalized to a percentage of torque produced at 100 Hz. Values reported as mean ± SE,* n* = 12. *Significantly greater compared to baseline.

There was no difference between conditions for torque generated during MVC and there was no difference in VA between conditions (Table 2). Furthermore, there was no difference in peak twitch torque, time to peak torque, or half relaxation time between conditions (Table 2). Absolute torque produced with 100 Hz stimulation during the torque‐frequency curve was not different between conditions (Fig. 5). Torque produced during the torque‐frequency curve was ~42% greater at 10 Hz with acute (46.6 ± 4.9% of 100 Hz torque; *P* = 0.0005) and chronic (47.2 ± 4.4%; *P* = 0.0002) BRJ supplementation compared to baseline (32.9 ± 2.6%) (Fig. 6). Furthermore, there was a trend for increased torque production at 20 Hz with acute (69.8 ± 5.5%; *P* = 0.09) and chronic (71.6 ± 4.8%; *P* = 0.08) BRJ supplementation compared to baseline (64.3 ± 4.8%).

**Table 2:** Maximum voluntary contraction (MVC) of the plantar flexors and twitch profile analysis of the plantar flexors following stimulation of the tibial nerve at baseline and following acute and chronic BRJ supplementation

	Baseline	Acute	Chronic
MVC (Nm)	91.3 ± 18.0	93.3 ± 12.7	90.8 ± 11.9
Voluntary activation (%)	96.4 ± 2.2	96.8 ± 4.0	96.3 ± 5.1
Peak twitch torque (Nm)	12.9 ± 2.8	13.1 ± 3.0	13.3 ± 2.5
Time to peak torque (msec)	208 ± 23	212 ± 23	212 ± 26
Half relaxation time (msec)	101 ± 15	100 ± 17	100 ± 16

Values reported as mean ± SD, *n* = 12.

## Discussion

The main novel finding from this study was that both acute and chronic BRJ supplementation did not reduce the O_2_ cost of submaximal exercise or improve aerobic TT performance in recreationally active females despite significant increases in plasma [${\text{NO}}_{3}^{-}$] and [${\text{NO}}_{2}^{-}$]. However, there was an increase in plantar flexor torque production with low frequency stimulation.

### Plasma ${\text{NO}}_{3}^{-}$ and ${\text{NO}}_{2}^{-}$, and serum estrogen and progesterone measures

Both acute and chronic dietary ${\text{NO}}_{3}^{-}$ supplementation elevated plasma [${\text{NO}}_{3}^{-}$] and [${\text{NO}}_{2}^{-}$] from baseline. The changes were slightly larger than previous human studies despite administration of a similar absolute dose of dietary ${\text{NO}}_{3}^{-}$ (Wylie et al. 2013; Whitfield et al. 2016). Since the baseline plasma [${\text{NO}}_{3}^{-}$] and [${\text{NO}}_{2}^{-}$] were similar to other studies (Wylie et al. 2013; Whitfield et al. 2016), this suggests that the lower BM of the female subjects in this study may account for the higher plasma [${\text{NO}}_{3}^{-}$] and [${\text{NO}}_{2}^{-}$]. However, we cannot rule out the possibility that sex differences may exist in dietary ${\text{NO}}_{3}^{-}$ clearance from the body. Previous work demonstrated that urinary ${\text{NO}}_{3}^{-}$, ${\text{NO}}_{2}^{-}$ and NO_x_ were not different between men and women following a low dose of dietary ${\text{NO}}_{3}^{-}$ (1.6 mmol) (Baião et al. 2016). This may suggest that women retain a greater proportion of the supplement, since the dose was larger per kg BM. Despite these slight differences in plasma concentrations, it is well accepted that ${\text{NO}}_{2}^{-}$ can be reduced to NO within the body, and therefore elevated plasma [${\text{NO}}_{2}^{-}$] is expected to increase NO availability which may elicit biological effects (Bailey et al. 2012). Importantly, the dose of dietary ${\text{NO}}_{3}^{-}$ administered in this study was high, especially in comparison to the female participants’ BM. This dose allowed for large increases in plasma [${\text{NO}}_{3}^{-}$] and [${\text{NO}}_{2}^{-}$] levels in these recreationally active females.

It has also been suggested that variations in female sex hormones throughout the menstrual cycle may influence endogenous NO production (Teran et al. 2002; Christin‐Maitre et al. 2011), skeletal muscle physiology and metabolism (Lundsgaard and Kiens 2014; Haizlip et al. 2015) as well as contractile properties (Sarwar et al. 1996). Therefore, all of the participants were actively using hormonal contraceptives which maintain female sex hormones at relatively constant levels throughout the menstrual cycle (Cicinelli et al. 1996). In accordance with this criterion, there was no change in serum estrogen or progesterone across trials suggesting that variability in female sex hormones was not a confounding issue throughout the study.

### Effects of BRJ supplementation on submaximal exercise economy and time trial performance

We did not observe a change in submaximal O_2_ uptake at 50% or 70% VO_2peak_ with either acute or chronic BRJ supplementation. This was corroborated by no change in HR or RPE throughout the submaximal exercise protocol. These findings are in contrast to previous acute and chronic BRJ supplementation studies utilizing recreationally active males, as they have consistently demonstrated improvements in submaximal exercise economy during cycling and running exercise (Bailey et al. 2009; Lansley et al. 2010; Vanhatalo et al. 2010). It is proposed that BRJ supplementation may reduce the O_2_ cost of submaximal exercise by improving skeletal muscle contractile efficiency (Bailey et al. 2010; Hernandez et al. 2012; Whitfield et al. 2017; Coggan and Peterson 2018) or mitochondrial efficiency (Larsen et al. 2011), although the latter has been challenged (Whitfield et al. 2016). The present findings suggest that sex differences may exist in the whole‐body responses of recreationally active athletes to acute and chronic BRJ supplementation. It is unclear whether the lack of effect in these recreationally active females is due to sex differences in skeletal muscle antioxidant capacity (Barp et al. 2002; Borrás et al. 2003), mitochondrial efficiency (Miotto et al. 2018), or fiber type (Haizlip et al. 2015) and contractile property differences (Wüst et al. 2008). Alternatively, it is possible that the use of hormonal contraceptives may have influenced the outcomes of this study. Future work is required to explore and elucidate the mechanisms underlying these potential sex differences, and the influence of hormonal contraceptives on the response to dietary ${\text{NO}}_{3}^{-}$ supplementation.

Furthermore, we did not observe a change in aerobic TT performance following acute or chronic BRJ supplementation. This is supported by our finding that acute and chronic BRJ supplementation did not alter submaximal exercise economy, therefore it is logical that we would not see differences in aerobic TT performance. This was corroborated by no difference in HR, PO or time elapsed during each 20% split of the TT indicating that acute and chronic dietary ${\text{NO}}_{3}^{-}$ supplementation did not impact any performance parameter throughout the TT. Importantly, all of the subjects in this study were successfully blinded to the experimental conditions, thereby strengthening our findings on aerobic TT performance.

The submaximal exercise economy and TT performance findings from this study are in contrast to our initial hypotheses. The first study providing insight that women may respond positively to acute and chronic dietary ${\text{NO}}_{3}^{-}$ supplementation was performed by Vanhatalo et al. (2010) who demonstrated a ~5% reduction in the O_2_ cost of moderate intensity exercise in a group of five males and three females following 2.5 h, 5 and 15 days of supplementation. Studies by Barclay et al. (2007) and Rienks et al. (2015) demonstrated significant reductions in the O_2_ cost of submaximal exercise following acute BRJ supplementation in sedentary and recreationally active females, respectively. However, these studies do not provide clear evidence for the positive effect of BRJ supplementation in recreationally active females. Bond et al. (2014) only reported graphical representations of their relative VO_2_ data and did not use a ${\text{NO}}_{3}^{-}$‐free PLA. Furthermore, these findings were in sedentary individuals, which does not accurately reflect the demographic in this study. Meanwhile, Rienks et al. (2015) evaluated exercise economy in recreationally active females by measuring VO_2_ during a 75 W recovery following a performance protocol. Although intriguing, these findings warranted a more robust and rigorous examination of the effects of BRJ supplementation on submaximal exercise economy. Lastly, three studies have been performed in elite female populations in which two demonstrate no beneficial effect with acute BRJ supplementation (Buck et al. 2015; Glaister et al. 2015) and one study shows chronic BRJ supplementation to improve aerobic and sprint swimming performance (Pospieszna et al. 2016). Although our submaximal exercise economy data demonstrate no effect of BRJ supplementation and are in contrast to other studies in non‐elite females (Bond et al. 2014; Rienks et al. 2015), we believe the data presented in this study provides a strong, well‐controlled and comprehensive investigation of acute and chronic BRJ supplementation on exercise economy and TT performance in recreationally active females.

### Influence of BRJ supplementation on contractile properties

It is important to highlight that previous research has shown that natural fluctuations in female sex hormones across the menstrual cycle influence skeletal muscle contractile properties (Sarwar et al. 1996). Furthermore, hormonal contraceptive use negates any influence of naturally fluctuating female sex hormones on skeletal muscle contractile properties (Sarwar et al. 1996). Therefore, we are confident that the observed contractile results are indicative of physiological changes following BRJ supplementation.

There were no differences in MVC, VA, or absolute electrically‐evoked torque produced at 100 Hz, which demonstrates the reproducibility of the electrical stimulation protocol across trials. Our torque‐frequency findings agree with other animal (Hernandez et al. 2012) and human studies (Haider and Folland 2014; Whitfield et al. 2017), where chronic BRJ supplementation increased low frequency torque production. This is the sensitive portion of the torque‐frequency curve and is physiologically relevant to the frequencies associated with voluntary skeletal muscle contraction (Oster and Jaffe 1980). Although Haider and Folland (2014) and Whitfield et al. (2017) used surface pad stimulation (vs. tibial nerve stimulation) to investigate the contractile properties of human quadriceps muscles, we found similar results in the plantar flexors by demonstrating increased torque production with low frequency stimulation (10 Hz). Furthermore, we expanded on the findings from these groups by reporting increased torque production with both acute and chronic BRJ supplementation at 10 Hz suggesting the role of post‐translational modifications on contractile proteins. This notion is supported in a recent review by Coggan and Peterson (2018) and by the principle that changes in protein content, which could explain the effect of BRJ supplementation do not occur merely 2.5 h following acute supplementation. Moreover, Whitfield et al. (2017) found no change in calcium‐handling protein content following 7 days chronic BRJ supplementation protocol.

Similar to Haider and Folland (2014) and Hoon et al. (2015), we did not find a difference in peak twitch torque, time to peak torque, and half relaxation time. These time parameters are indirect measures for calcium‐handling properties suggesting no difference in calcium release or reuptake in response to skeletal muscle contraction following acute and chronic BRJ supplementation in recreationally active females. Half relaxation is pivotal in determining summation and ultimately skeletal muscle torque production. Therefore, shortened half relaxation relative to time to peak torque suggests increased rates of calcium reuptake, which may translate to decreased unfused tetanic skeletal muscle torque production through the blunting of summation. The findings from this study are logical as we demonstrate increased low frequency torque production with acute and chronic BRJ supplementation without alterations to calcium release or reuptake.

Taken together, it is possible that BRJ supplementation improves myosin ATPase function by increasing the torque produced per ATP consumed. However, in absolute terms our findings indicate a 3 to 5 Nm increase in torque production in a muscle that is not critical to torque production during cycling. Future work needs to investigate whether a similar response is elicited in the quadriceps and gluteal regions in recreationally active females as these are the major torque producing muscles in cycling.

### Individual responders

A responder was arbitrarily defined as a subject who demonstrated a ≥3% reduction in VO_2_ following BRJ supplementation compared to PLA. This is in accordance with previous research (Wylie et al. 2013) and is outside the calculated CV for VO_2_ at 50% VO_2peak_ (2.4%) and 70% VO_2peak_ (2.0%). Interestingly, 25% of the subjects were defined as responders to Acute BRJ supplementation and 42% were defined as responders to Chronic BRJ supplementation at 50% VO_2peak_. At 70% VO_2peak_, 25% of the subjects were defined as responders to Acute BRJ supplementation and 33% to Chronic BRJ supplementation.

Across the two studies, one “responder” stood out in the group of seven recreationally active females that completed both protocols. This subject demonstrated a reduced O_2_ cost of exercise at 50% VO_2peak_ acutely (49 mL/min) and chronically (10 mL/min), as well as a reduced O_2_ cost of exercise at 70% VO_2peak_ acutely (69 mL/min) and chronically (85 mL/min). The same subject also demonstrated an improvement in aerobic TT performance following acute (92 sec) and chronic (106 sec) BRJ supplementation and in skeletal muscle torque production at 10 Hz with acute (40%) and chronic (18%) BRJ compared to baseline.

This finding is important because it demonstrates that some individuals do respond to acute and chronic BRJ supplementation. Furthermore, it is possible that there is a link between increased skeletal muscle torque production, decreased O_2_ cost of exercise, and improved aerobic TT performance, even if in one subject. In the current study, this responder could not be identified by baseline plasma [${\text{NO}}_{3}^{-}$] or [${\text{NO}}_{2}^{-}$] or by rises in plasma [${\text{NO}}_{3}^{-}$] or [${\text{NO}}_{2}^{-}$] following supplementation. Furthermore, this subject did not demonstrate a different fitness status, training volume, or dietary habits from the group. Future research is required to understand individual variability in the response to dietary ${\text{NO}}_{3}^{-}$ supplementation.

### Limitations and future directions

In the current study, the CV for VO_2_ between the second familiarization trial, acute placebo and chronic placebo trial, where there was no effect of condition was 2.4% at 50% VO_2peak_ and 2.0% at 70% VO_2peak_. Therefore, it is possible that reductions in VO_2_ with BRJ supplementation smaller than these CVs were not detected.

The present study used a 4 kJ/kg time trial to assess exercise performance. This style of time trial has been validated to have greater ecological validity compared to time to exhaustion protocols (Currell and Jeukendrup 2008). However, this may not be a valid measure of performance in recreationally active individuals who do not train for TT performance. We ensured that each participant was adequately familiarized with the TT protocol twice and that there was less than 5% difference in TT completion before they graduated to the experimental trials. Interestingly, the CV for TT performance between the second familiarization trial, acute placebo and chronic placebo trial, where presumably there was no effect of condition was 4.2%, which agrees with Currell and Jeukendrup (Currell and Jeukendrup 2008) suggesting <5% variability in TT performance for trained individuals. This indicates that this protocol had high repeatability despite the recreational status of the participants. Lastly, we measured serum [estradiol] and [progesterone] and effectively demonstrated that females using hormonal contraceptives do not produce their own endogenous estrogen and progesterone. However, due to financial constraints, we did not measure ethinyl estradiol and the many variations of synthetic progesterone that comprise hormonal contraceptives. Therefore, despite demonstrating a high level of control for natural hormone production throughout the menstrual cycle while using hormonal contraceptives, we were unable to confirm in our hands that synthetic hormone levels remain constant across the menstrual cycle while using hormonal contraceptives. However, this has been demonstrated in previous literature (Christin‐Maitre et al. 2011). Furthermore, it remains to be determined if synthetic female sex hormones influence endogenous NO production (Merki‐Feld et al. 2002; Cherney et al. 2007) or skeletal muscle metabolism (Merki‐Feld et al. 2002).

Following the completion of this study, a number of questions remain unanswered regarding sex differences that may exist with BRJ supplementation. Future work should include a study where subjects are tested for the VO_2_ effects of BRJ while sedentary and then following an endurance training regime. It is possible that sedentary females may demonstrate a reduction in the O_2_ cost of submaximal exercise following BRJ supplementation as previously reported by Bond et al. (2014), however this effect may be lost in recreationally active and trained female populations due to enhanced endogenous NO production, O_2_ delivery, and contractile efficiency. Second, future work should directly compare the responses to acute and chronic BRJ supplementation in recreationally active male and female populations. Muscle biopsies should be obtained to delve into the mechanisms underpinning the potential sex differences that may exist. Specifically, potential sex differences in skeletal muscle antioxidant capacity, calcium‐handling protein function, and mitochondrial efficiency should be investigated. Furthermore, recent evidence identifies skeletal muscle as a storage reservoir for ${\text{NO}}_{3}^{-}$ following acute sodium ${\text{NO}}_{3}^{-}$ supplementation (Nyakayiru et al. 2017b). Therefore, it is possible that sex differences in absolute skeletal muscle mass and distribution, and ultimately ${\text{NO}}_{3}^{-}$ storage, may contribute to the divergent responses to dietary ${\text{NO}}_{3}^{-}$ supplementation. Clearly, incorporating females into BRJ research is in its infancy and there are a number of avenues yet to be explored.

## Conclusions

Both acute and chronic dietary ${\text{NO}}_{3}^{-}$ supplementation successfully increased plasma ${\text{NO}}_{3}^{-}$ and ${\text{NO}}_{2}^{-}$ levels in recreationally active females. However, this did not translate to a reduction in the O_2_ cost of submaximal exercise and did not improve aerobic TT performance, despite increased plantar flexor torque production with low frequency stimulation at 10 Hz. However, even if this occurred in the muscles predominantly involved in cycling, this improvement may not be significant enough to translate into reductions in whole‐body VO_2_ in recreationally active females. Future research should investigate potential sex differences that may exist in the responses to acute and chronic dietary ${\text{NO}}_{3}^{-}$ supplementation.

## Conflict of Interest

No conflicts of interest, financial or otherwise are declared by the authors.
